# Impact and prevalence of comorbidities and complications on the severity of COVID-19 in association with age, gender, obesity, and pre-existing smoking: A meta-analysis

**DOI:** 10.37796/2211-8039.1429

**Published:** 2024-03-01

**Authors:** Soulandi Djorwé, Amale Bousfiha, Néhémie Nzoyikorera, Joseph Nyandwi, Bellamine Kawthar, Abderrahim Malki

**Affiliations:** a Laboratory of Physiopathology and Molecular Genetics, Faculty of Sciences Ben M’Sik, Hassan II University of Casablanca (Morocco), Avenue Cdt Driss El Harti, PB 7955, Sidi Othman, Casablanca, Morocco; b Bourgogne Laboratory of Medical and Scientific Analysis, 136, Residence Belhcen, Bd Bourgogne, Casablanca, Morocco; c National Reference Laboratory, National Institute of Public Health, Burundi; d Higher Institute of Biosciences and Biotechnology, Mohammed VI University of Health Sciences (UM6SS), Casablanca, Morocco; e Laboratory of Microbial Biotechnology and Infectiology Research, Mohammed VI Center for Research & Innovation, Rabat, Mohammed VI University of Health Sciences (UM6SS), Casablanca, Morocco; f Département de Médecine, Faculté de Médecine, Université du Burundi, Burundi; g Ministère de la Santé Publique et de la Lutte contre le Sida, Institut National de Santé Publique de Bujumbura, Burundi

**Keywords:** Severe COVID-19, Non-severe COVID-19, Comorbidities, Complications, Risk factors

## Abstract

**Background:**

COVID-19 patients usually present multiple comorbidities and complications associated with severe forms of SARS-CoV-2 infection. This study aimed to assess the risk factors and prevalence of comorbidities and complications contributing to the severity of COVID-19.

**Methods:**

This meta-analysis was performed according to PRISMA guidelines. We searched various databases, including PubMed, Google Scholar, and Scopus (between 2020 and 2023), for eligible studies for this meta-analysis.

**Results:**

Thirty-three studies were eligible, including 85,812 patients, of which 36 % (30,634/85,812) had severe disease, whereas 64 % (55,178/85,812) had non-severe disease. Severe cases were potentially correlated with the following factors: gender (male) (odd ratio (OR) = 1.52, 95 % CI: 1.34–1.73), advanced age (OR = 3.06, 95 % CI: 2.18–4.40) pre-existing smoking (OR = 1.33, 95 % CI: 1.01–1.75), obesity (OR = 2.11, 95 % CI: 1.47–3.04), diabetes (OR = 1.81, 95 % CI: 1.35–2.43), hypertension (OR = 2.22, 95 % CI: 1.72–2.87), coronary heart disease (OR = 2.17, 95 % CI: 1.42–3.31), CKD (OR = 2.27, 95 % CI: 1.26–4.06), COPD (OR = 1.95, 95 % CI: 1.22–3.09), malignancy (OR = 1.63, 95 % CI: 1.07–2.49) and cerebrovascular disease (OR = 2.76, 95 % CI: 1.63–4.62). All these comorbidities were significantly higher in the severe COVID-19 group compared with the non-severe COVID-19 group. In addition, the most severe complications were associated with shock (OR = 28.08, 95 % CI: 3.49–226.03), ARDS (OR = 13.09, 95 % CI: 5.87–29.18), AKI (OR = 16.91, 95 % CI: 1.87–152.45) and arrhythmia (OR = 7.47, 95 % CI: 2.96–18.83). However, these complications were the most likely to prevent recovery in patients with severe affections compared with non-severe affection groups.

**Conclusion:**

All the comorbidities and complications listed above are more likely to cause severe forms of COVID-19 in some patients and hinder recovery. They are therefore risk factors to be controlled to minimize the undesirable effects of the disease.

## 1. Introduction

The COVID-19 pandemic, caused by severe acute respiratory syndrome coronavirus 2 (SARS-CoV-2), has caused high morbidity and mortality around the world. More than 0.67 billion people have been infected and more than 6.8 million deaths around the world [1,2]. The clinical presentation of SARS-CoV-2 infection is very wide, ranging from asymptomatic infection in the first phases of infection to severe symptomatic forms of infection, sometimes implying dyspnea, organ dysfunction and, in the worst case, death ensues [2].

Several studies have reported that there are many clinical risk factors associated with the development of severe or even potentially fatal forms of the disease (COVID-19). These risk factors included demographic characteristics such as gender, advanced age, smoking history; comorbidities such as diabetes mellitus, hypertension, cardiovascular diseases, and chronic obstructive pulmonary disease (COPD), as well as complications such as acute respiratory distress syndrome (ARDS), septic shock, and, in the most extreme cases, neurological complications [3]. In addition, recent studies have highlighted the syndemic concept in the severity of COVID-19 [4,5]. Defined (syndemic) as two or more health conditions or diseases which, by their synergy, aggravate the consequences of these diseases on a sick person, specifically, an unhealthy lifestyle behaviors (i.e., physical inactivity, poor diet, increased sedentariness, obesity etc.)—chronic diseases—COVID-19 syndemic [4,5]. Indeed, emerging evidence in the scientific literature has suggested that some regions of the world suffer from the syndemic rather than the pandemic [4]. This syndemic would be one of the main causes of hospitalizations and high mortality worldwide during the COVID-19 pandemic [4–6].

Nevertheless, a good understanding of the risk factors associated with COVID-19 severity is useful for public health and clinicians to implement appropriate preventive measures and guidance of emerging treatment protocols to better identify patients at high risk of severe COVID-19 for priority treatment to prevent disease progression and unfavorable outcomes [7].

The aim of this study was to exhaustively evaluate the risk factors and prevalence of demographic characteristics, comorbidities, and complications associated with the severity of COVID-19 and the correlation between these risk factors.

## 2. Methods

The Preferred Reporting Items for Systematic Review and Meta-Analysis (PRISMA) statement (Supporting information: S1 PRISMA 2020 checklist) and the statement by the Meta-analysis of Observational Studies in Epidemiology (MOOSE) group were employed to design and describe this study [8,9].

### 2.1. Search strategy

To minimize bias, two investigators (S.D and NN) independently examined the data (titles, abstracts, and extraction of potentially eligible articles). Eligible articles were stored in a “Zotero” citation manager. Literature searches were performed using the international databases PubMed Central, Google Scholar, and Scopus, using the following search terms:

“SARS-CoV-2” OR “COVID-19” AND “Severity and non-severity of COVID-19” OR “Severity of COVID-19” OR “Intensive Care Unit” OR “clinical features of COVID-19”. The articles selected for this study are those published between 2020 and 2023.

### 2.2. Selection criteria

Studies fulfilling the following selection criteria were included in the meta-analysis: - Inclusion criteria: (1) study type: case series, cohort study, or prospective study in relation to the severe form of COVID-19; (2) COVID-19-positive patients with comorbidities and associated studies reporting complications following the severity of COVID-19; (3) randomized clinical trials, observational studies, and case series involving ≥70 patients, written in English or French.

- Exclusion criteria were: (1) systematic reviews and meta-analyses; (2) unpublished articles; (3) studies with sample sizes ≤70 patients; (4) insufficient or incomplete data; (5) animal experiment reports, and (6) pediatric reports.

### 2.3. Quality assessment

The evaluation of the quality of studies included in this meta-analysis was based on the Newcastle-Ottawa Scale (NOS) [10]. The main elements include: selection (representativeness of the exposed cohort, selection of the nonexposed cohort, Ascertainment of exposure, demonstration that the outcome of interest was not present at the start of the study); comparability (comparability of cohorts on the basis of design or analysis); and outcome (assessment of outcome, follow-up time, Adequacy of follow-up of cohorts). The quality score ranges from 0 to 10 stars, with a score ≥7 stars indicating high-quality articles (Table 1).

### 2.4. Statistical analysis

All statistical tests were performed using Review Manager (RevMan) version 5 (Cochrane Collaboration, Copenhagen, Denmark) [11]. Odds ratios (ORs) were calculated for dichotomous variables, while standardized mean differences (SMDs) and 95 % confidence intervals (CIs) were calculated for continuous data. The Mantel-Haenszel random-effects model was used to calculate effect sizes. Heterogeneity was estimated using the chi-square test and the Higgins I^2^ test. Z-score was calculated to detect the overall effect, with significance set at P < 0.05. Publication bias was examined using funnel plots. The 95 % CI in the funnel plots is based on the hypothesis that all studies have only one true effect. Consequently, the 95 % CI in the fixed-effects model (based on this hypothesis) represents the expected distribution of studies in the absence of heterogeneity and bias. The random-effects model, on the other hand, supposes that these studies have different true effects, and there is therefore no need to add a 95 % CI. For all analysis, significant levels were two-tailed, and p < 0.05 was considered significant for OR and SMD summarized. Population attributable risk fractions (PAF) for COVID-19-positive patients for the effect of the outcome of different comorbidities were also calculated for all groups. The formula used to calculate PAF was:


$$\text{PAF}={\text{P}}_{\text{1}}\;(\text{OR-}1)/\text{OR}$$

P 1 = proportion of risk factors in the population and OR = odds ratio [12].

For continuous variables for which SMD has been calculated, the odd ratio is determined according to the equation: ln (OR) = π/√3 X SMD [13].

## 3. Results

### 3.1. Study selection and characteristics

**Fig. 1:**
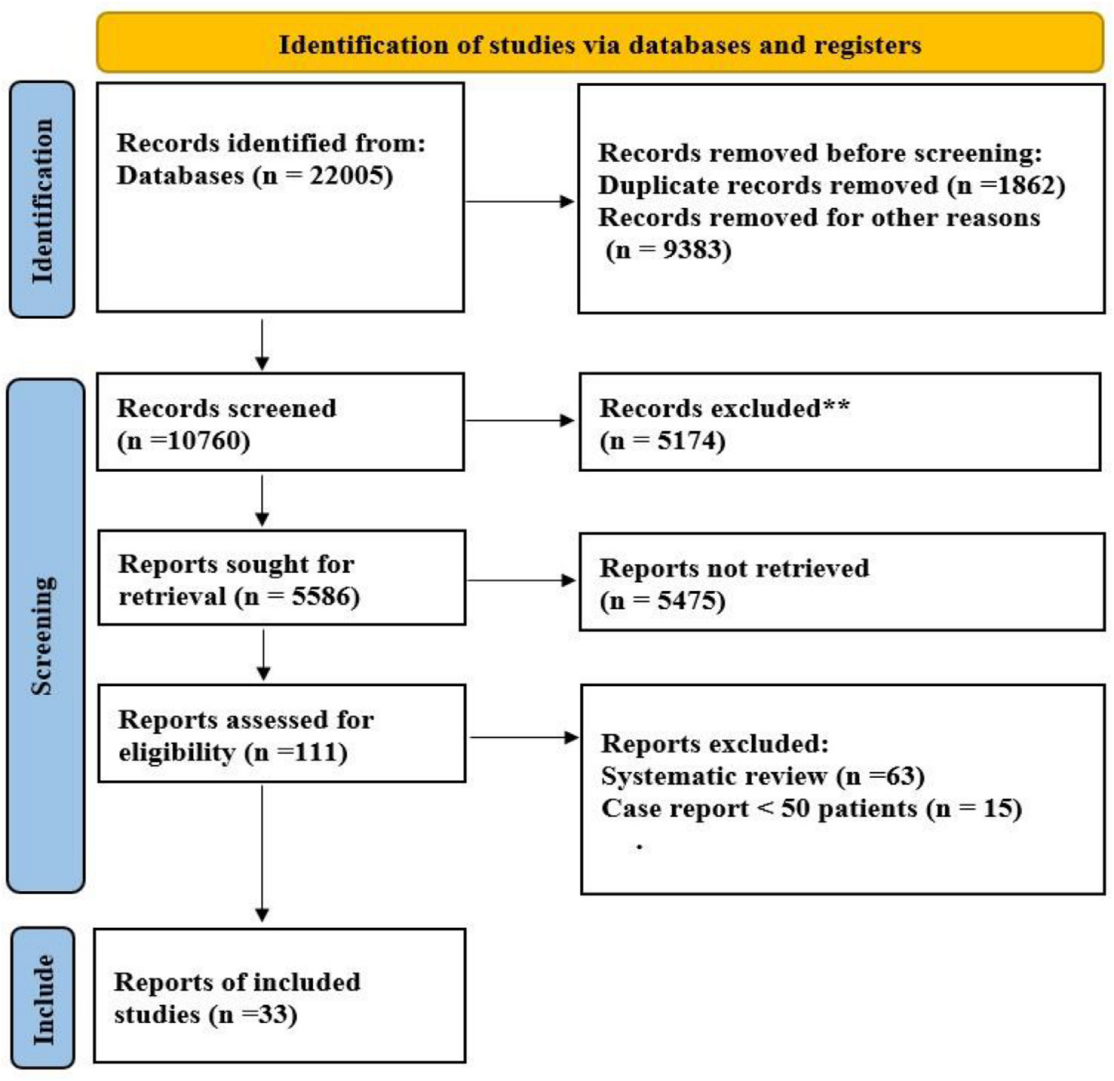
PRISMA flow diagram of study selection.

Fig. 1 shows the flow chart for study selection according to PRISMA guidelines. According to the predefined search strategies, a total of 22,005 articles were initially identified, of which 21,972 were excluded following evaluation of the eligibility criteria. COVID-19 patients were divided into severe and non-severe groups. Study characteristics are shown in Table 1.

A total of 85,812 patients were included in this meta-analysis. The characteristics of all studies included are listed in Table 1. The rate of severe cases was 36 % (30,634/85,812), while the rate of non-severe cases was 64 % (55,178/85,812).

### 3.2. Quality of the included studies

The results of the quality assessment of all the studies included in this meta-analysis are presented in Table 2. The majority of studies were high-quality, with the exception of two studies with a NOS score of 6. The NOS scores of the thirty-one other studies with high quality were distributed as follows: nine studies had a NOS score of 7, nine other studies had a NOS score of 8, and thirteen studies had a NOS score of 9.

### 3.3. Summary of the meta-analysis for primary outcomes

In Table 3, sixteen risk factors for COVID-19 have been listed in three categories. These are demographic characteristics, comorbidities, and complications that have an impact on the severity of COVID-19.

### 3.4. Publication bias

**Fig. 2:**
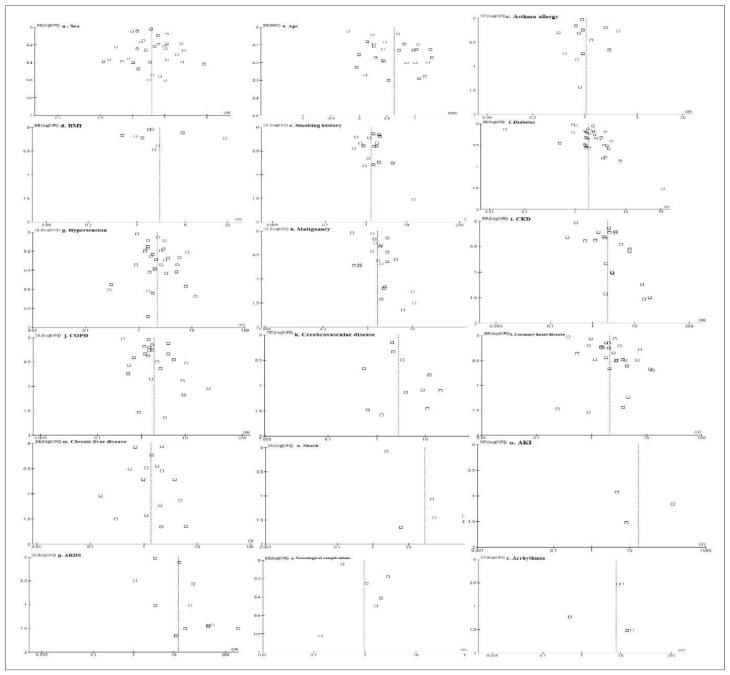
Funnel plot representing publication bias of each meta-analysis: (a) sex, (b) age, (d) smoking history, (e) BMI, (e) asthma/allergy, (f) diabetes, (g) hypertension, (h) malignancy, (i) CKD, (j) COPD, (k) cerebrovascular disease, (l) coronary heart disease, (m) chronic liver disease, (n) shock, (o) acute kidney injury (AKI), (p) acute respiratory distress syndrome (ARDS), (q) neurological complications and (r) arrhythmia. *SE (log [OR]) = Standard error multiplied log scale of odd ratio (OR).

In order to estimate publication bias in addition to heterogeneity (chi-square test and Higgins’ I**^2^** test), funnel plots were generated (Fig. 2). Funnel plots of demographics, comorbidities, and complications in this meta-analysis showed that nearly all the funnel plots were asymmetrical with the exception of chronic liver disease, cerebrovascular disease, asthma/allergy, and AKI, implying that the publication bias existed to some extent.

### 3.5. Demographic characteristics

**Fig. 3:**
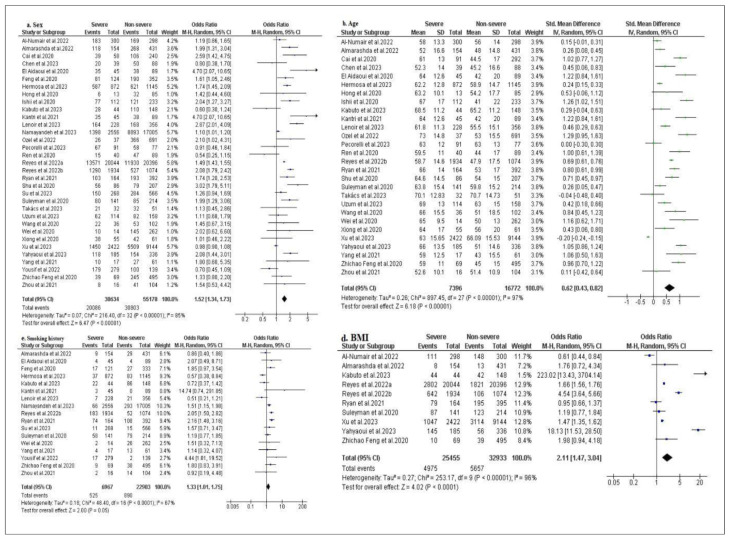
Forest plots comparing demographic characteristics between severe and non-severe groups. (a) sex, (b) age, (d) smoking history, (e) BMI and severe COVID-19 disease.

Demographic characteristics in this study included gender, age, pre-existing smoking, and body mass index (BMI ≥25–30 kg/m^2^) (BMI is the most useful measure of overweight and obesity in a population, because in adults, the scale is the same regardless of the subject’s gender or age (according to World Health Organization (WHO), there is overweight when BMI ≥25 kg/m^2^; and there is obesity when BMI is ≥30 kg/m^2^)). The results of the analysis of demographic characteristics are presented in Fig. 3. All thirty-three studies reported gender differences (men were taken as reference group in this study), and the result of our analysis showed that men were more likely to have severe disease than women (OR = 1.52, 95 % CI:1.34–1.73; I^2^ = 85 %, p < 0.001) [14–46]. In this study, age was statistically significant (SMD = 0.62, 95 % CI: 0.43–0.82 of which OR = 3.06, 95 % CI: 2.18–4.40; I^2^ = 97 %; p < 0.001) [14–25,27–46]. Of all articles included in this study, the mean age of patients with severe affection was higher than that of patients with non-severe affection, except for the studies of Takacs et al., 2023 and Xu et al., 2023 [36,41]. Patients’ pre-existing smoking status was positively correlated with severe coronavirus disease (OR = 1.33, 95 % CI: 1.01–1.75; I**^2^** = 67 %, P < 0.001) [15,18–20,23–26,31,32,34,35,39,43–46]. Analyses based on BMI (BMI ≥25–30 kg/m2) revealed that overweight and obesity (overweight if BMI ≥25 kg/m^2^; and obesity if BMI ≥30 kg/m^2^) were higher in patients with the severe form of the disease compared to the group of patients with non-severe affection (OR = 2.11, 95 % CI: 1.47–3.04; I**^2^** = 96 %; P < 0.001) [14,15,23,30–32,35,41,42,45]. Fig. 3 shows forest plots of the meta-analysis of the association between demographic characteristics of the severity and non-severity of COVID-19 disease: (a) gender, (b) age, (d) smoking history, and (e) BMI.

### 3.6. Comorbidities

**Fig. 4:**
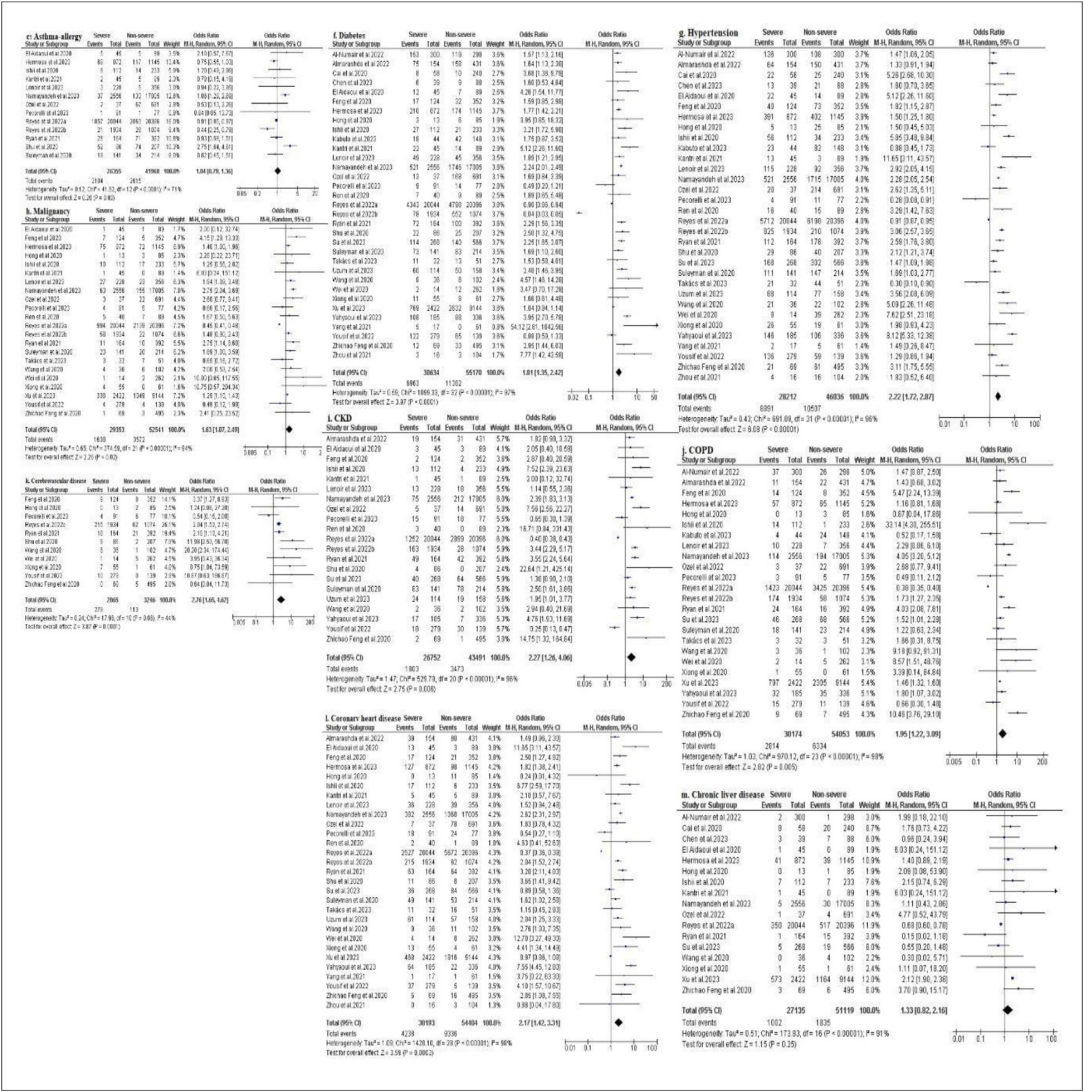
Forest plots of the meta-analysis of the association between comorbidities in patients with severe and non-severe COVID-19 disease. (e, f, g, h, i, j, k, l, m) forest plots of the association between (e) asthma/allergy, (f) diabetes, (g) hypertension, (h) malignancy, (i) CKD, (j) COPD, (k) cerebrovascular disease, (l) coronary heart disease, and (m) chronic liver disease.

In this study, Fig. 4 shows the potential association between nine comorbidities and the risk of severe COVID-19. In comparison with patients with a non-severe form of COVID-19 disease, there was a higher risk in patients with the severe form of COVID-19 with several comorbidities associated. Our results showed that cerebrovascular disease had the highest OR value of 2.76 (95 % CI: 1.65–4.62; I^2^ = 44 %; p = 0.06) [19,21,28,31–33,38–40,44,45] despite the fact that no significant difference between patients with severe and non-severe cerebrovascular disease was reported after analysis. This risk is followed by chronic kidney disease (CKD), with an OR of 2.27 (95 % CI: 1.26–4.06]; I^2^ = 96 %; P < 0.001) [15,18,19,22,24, 26–35,37,38,42,44,45], hypertension with an OR of 2.22 (95% CI: 1.72–2.87; I^2^ = 96 %; p < 0.001) [14–40,42–46] and coronary heart disease with an OR of 2.17 (95 % CI: 1.42–3.31; I**^2^** = 98 %; p < 0.001) [15,18–22,24–46]. Other significant judgment criteria for risk factors of the comorbidities involved in the severe form of COVID-19 included chronic obstructive pulmonary disease (COPD) (OR: 1.95, 95% CI: 1.22–3.09; I**^2^**=98%, p < 0.001) [14,15,19–23,25–28,30–32,34–36,38–42, 44,45], diabetes mellitus (OR=1.81, 95% IC: 1.35–2.43; I^2^=97 %, p<0.001) [14–46], malignancy (OR=1.63, 95 % CI: 1.07–2.49; I**^2^** = 94 %; p < 0.001) [18–22,24–32,35,36,38–41,44,45], Chronic liver disease (CLD)with an OR of 1.33 (95% CI: 0.82–2.16; I**^2^**=91%, p < 0.001) [14,16–18,20–22,24,26,27,30,32,34,38,40, 41,45] and Asthma/allergy (OR = 1.04, 95 % CI: 0.79–1.36; I**^2^** = 71 %, p < 0.001) [18,20,22,24–28, 30–33,35]. However, there were fewer patients with asthma or allergies in the severe COVID-19 group compared to the non-severe COVID-19 group with a significant number of patients with asthma or allergies.

### 3.7. Complications

**Fig. 5:**
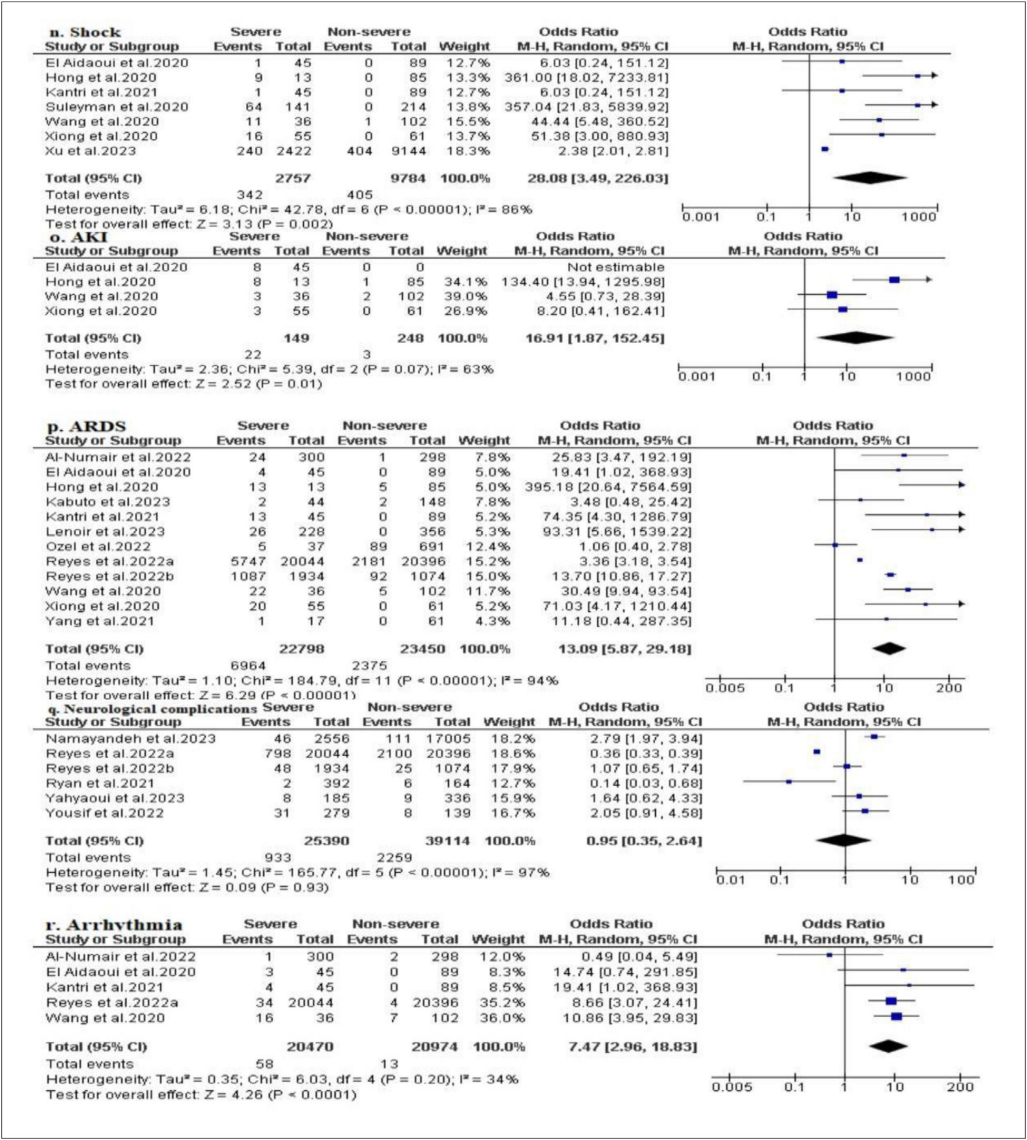
Forest plots of complications reported in severe and non-severe groups of patients with COVID-19: (n) shock, (o) acute kidney injury (AKI), (p) acute respiratory distress syndrome (ARDS), (q) neurological complications, (r) arrhythmia.

In Fig. 5, our results indicate that shock with an OR of 28.08 (95 % CI: 19,99–78,41; I**^2^** = 86 %, p < 0.001) [18,21,24,35,38,40,41], acute kidney injury (AKI) with OR = 16.91 (95 % CI: 1.87–152.45; I**^2^** = 63 %; p = 0.07) [18,21,38,40] and acute respiratory distress syndrome (ARDS) with OR = 13.09 (95 % IC: 5.87–29.18; I**^2^** = 94 %, P < 0.001) [14,18,21,23–25,27,30,31,38,40,43] were the principal complications associated with severe and life threatening disease in COVID-19 patients. In addition to the aforementioned complications, cardiac rhythm disturbance (arrhythmia) (OR = 7.47; 95 % CI: 2.96–18.83; I**^2^** = 34 %, p = 0.20) [14,18,24,30,38] was one of the complications associated with the severity of COVID-19 disease in some patients, although the result was not statistically significant. Despite the fact that the studies we examined provided insufficient evidence to support other findings of complications other than shock, acute renal failure (AKI), acute respiratory distress syndrome (ARDS), and arrhythmia, the data that we have are very consistent in severe cases of COVID19. However, neurological complications with an OR of 0.95 (95 % CI: 0.35–2.64; I^2^ = 97 %, p < 0.001) did not show any potential risk of complication in the group of patients with severe COVID-19 disease [26,30–32,42,44].

### 3.8. Population attributable risk

In this meta-analysis, we calculated the proportion of risk attributed to the population infected with SARS-CoV-2. The Population Attributable Fraction (PAF) represents the proportion of cases in the population that can be attributed to exposure to risk factors associated with severe infections of the coronavirus disease (COVID-19). In this study, the PAFs ranged from 0.10 % to 18.47 % (Table 4). The estimated attributable fraction for COVID-19 patients was 18.47 % for acute respiratory distress syndrome (ARDS), 14.29 % for hypertension, 9.47 % for body mass index (obesity), 9.39 % for diabetes, and 8.62 % for coronary heart disease. It is estimated that reducing the prevalence of ARDS, hypertension, BMI, diabetes, and coronary heart disease could have prevented up to 8.62 %–18.47 % of severe cases.

## 4. Discussion

To our knowledge, this is one of the most comprehensive studies demonstrating the impact of various comorbidities and complications on the severe cases of COVID-19, as well as estimating the prevalence of these risk factors. Identifying potential risk factors involved in severe coronavirus disease is crucial for public health professionals to better target high-risk patients requiring prioritized treatment to prevent unfavorable disease outcomes [7]. In this study, potential risk factors contributing to severe form included demographic characteristics such as age, gender, obesity (BMI ≥25–30 kg/m^2^) and smoking history, as well as comorbidities and complications listed in Table 3. The different risk factors reported in this meta-analysis are predictive of a severe and critical form of the disease, requiring patients’ admission to intensive care units (ICU). Our analysis has shown that the association between age, gender (male), obesity, and smoking status appeared with numerous comorbidities as risk factors for severe COVID-19 [47,48]. Indeed, several studies have reported that advanced age is associated with the severity of COVID-19, primarily due to physiological changes accompanying aging, a weakened immune system and underlying health issues [7,49,50]. In agreement with these studies, the advanced age of patients in our study appeared strongly as a potential risk factor associated with the severe form of COVID-19 compared to non-severe COVID-19 patients (SMD = 0.62; OR = 3.06, P < 0.001), and the result was statistically significant. However, age alone does not explain the variability in disease severity. In agreement with the results from the general population in this study, it was evident that the elderly had a higher prevalence of comorbidities including diabetes (OR = 1.81), hypertension (OR = 2.22), and COPD (OR = 1.95) as observed in our study (Fig. 4), while complications including ARDS (OR = 13.09, PAF = 18.47 %), shock (OR = 28.08, PAF = 5.78 %), AKI (OR = 16.91, PAF = 5.64 %) and arrhythmia (OR = 7.47, PAF = 0.17 % %) were the main obstacles to recovery in patients with the severe form of COVID-19 (Fig. 5 and Table 3). In addition, our study supports the hypothesis of some studies which have reported that, cancer (OR = 1.63), CKD (OR = 2.27), cerebrovascular disease (OR = 2.76), and coronary heart disease (OR = 2.17), are also associated with the severity of COVID-19 [49,51,52]. These risk factors increase the risk of admission to intensive care units, and could be life-threatening [49,51,52]. In contrast to some studies, our study reported several comorbidities [47,52–54]. Interestingly, previous studies such as that of Ross Arena and his colleagues have indicated that unhealthy lifestyle behaviors, comorbidities, and COVID-19 syndemic in severe cases raise the question of a complex interaction of health factors, acting synergistically to worsen the overall consequences of the disease [4–6]. It is reported that physical inactivity can contribute to obesity, which in turn can increase the risk of diabetes and other noncommunicable diseases (NCDs) [4–6]. Our study supports this hypothesis, which is consistent with our results showing that patients with comorbidities such as obesity, diabetes, and cardiovascular disease were at high risk of developing the severe form of COVID-19. Therefore, raising awareness of healthy lifestyles could strengthen human resilience and could protect some people against certain noncommunicable diseases as well as health deterioration due to viral infection [4–6].

Advanced age and comorbidities such as diabetes, hypertension, and asthma have been found to have a significant impact on the health of COVID-19 patients. Previous meta-analysis studies have shown that pre-existing diabetes, hypertension, obesity, and smoking were associated with severe cases of COVID-19 and higher mortality, representing up to 30 % of cases [47]. Therefore, it is essential to pay particular attention to obese elderly patients with a history of smoking, as well as those with comorbidities or complications such as shock, ARDS, AKI, and arrhythmia because they are likely to develop a severe form of COVID-19 [47]. According to some reports, the proportion of COVID-19 cases is approximately equal between the genders [55], but men are more likely to develop a severe form of the disease [52]. Our analysis is in agreement with the fact that male gender (OR = 1.65) is one of the risk factors associated with the severity of COVID-19 unlike female gender [56]. Nevertheless, it is reported that the severity of COVID-19 which mainly affects elderly men is strongly associated with frequent consumption of tobacco [56]. Moreover, certain genes regulating the immune system are located on the X chromosome, of which women possess two copies. These genes located on the X chromosome contain the largest number of genes regulating the immune system in the entire human genome, theoretically conferring women a double advantage in stimulating an effective and rapid immune response [57]. In addition, the female hormones estrogen and progesterone would boost women’s immunity against severe COVID-19, while, lower expression of the androgen receptor (AR) in women might contribute to reduced vulnerability to complications like acute respiratory distress syndrome [58,59]. Angiotensin-converting enzyme 2 (ACE2) an essential receptor for cell entry of SARSCoV-2 is known to be influenced by sex hormones and is present in greater quantity in men than in women, which could increase sensitivity to SARS-CoV-2 [60,61]. In addition, the host transmembrane serine protease 2 (TMPRSS2) also plays an important role in the entrance of SARS-CoV-2 into cells. Its expression is associated with an increase in androgen receptors [62,63]. Our study showed that pre-existing diabetes was a risk factor (OR = 1.81) associated with severe disease in patients with COVID-19 compared to patients with non-severe forms of the disease. This finding is in agreement with several related studies [64,65]. Diabetic patients suffer from altered immunological and inflammatory pathways after hyperglycemia, which could make them more susceptible to developing a severe COVID-19 infection [66]. In other words, high blood glucose levels in diabetic patients could make them more vulnerable to severe COVID-19 infection. On the other hand, COVID-19 infection can influence glucose levels and worsen outcomes in diabetic patients. The severe form of COVID-19 can stimulate the liberation of stress hormones such as cortisol and adrenalin, temporarily raising blood glucose levels by stimulating glucose production in the liver [66]. In addition, the systemic inflammation associated with infection can disrupt insulin regulation, leading to insulin resistance and increased glucose levels [66], therefore, this suggests a bidirectional association between diabetes/glycemia and COVID-19 [67]. In addition to the comorbidities previously mentioned, obesity is identified as another metabolic comorbidity associated with severe COVID-19. We considered obesity as a risk factor on the basis of this meta-analysis (OR = 2.11) with a prevalence of 9.47 % associating obesity with risk factors for severe COVID-19. Obesity is implicated in significant changes in the distribution of immune cells in adipose tissue, with a decrease in regulatory T cells (Treg), Th2 cells, and M2 macrophages, as well as a decrease in M1 macrophage cells and an increase in CD8+ T lymphocytes, hampering immune defense and T cell activity [47,66,68]. The high level of ACE2 receptor expressed in adipocytes may transform adipose tissue into a viral vector, which could facilitate the spread of SARS-CoV-2 to other organs [69].

Hypertension was one of the major predominant factors for disease severity of COVID-19. It is a chronic disease that generally occurs in the elderly. In the severe form of COVID-19, there is a strong correlation between hypertension and age, on the one hand, and coronary heart disease on the other hand. This correlation could explain the high prevalence of hypertension in patients with severe COVID-19 [70,71]. Considered the leading reversible risk factor for cardiovascular morbidity and mortality, contributing to the development of vulnerable cardiovascular areas and acute myocardial affections favoring severe and fatal forms of COVID-19 [72]. Recent reports have shown that the prevalence of hypertension in patients with COVID-19 is considerable, ranging from 9.6 % to 40.8 % [70]. According to our results, most patients with severe COVID-19 were older than those with non-severe COVID-19 and the prevalence of hypertension in our study was 14.29 % with an OR of 2.22 and with an OR of 2.17 for coronary heart disease and arrhythmic complications (OR = 7.47) implicated in the worsening of COVID-19 in patients. These results were consistent with related studies [70,71]. SARS-CoV-2 infection is therefore associated with a high inflammatory load that can induce vascular inflammation, myocarditis, and cardiac arrhythmias. This suggests that myocardial and vascular damage may be direct or indirect after SARS-CoV-2 infection through myocarditis and endothelial dysfunction [73]. Furthermore, in subjects with severe COVID-19, systemic inflammation, elevated cytokine levels and hypercoagulability could contribute to atherosclerotic plaque rupture [71]. In addition, sympathetic hyperactivity and hypoxemia can lead to myocardial ischemia and ventricular dysfunction by disturbing the balance between the heart’s oxygen supply and consumption [71]. In normal physiological conditions, the antagonistic effects of ACE 1 and ACE 2 maintain homeostasis. However, in patients with severe coronavirus disease, angiotensin-1 is converted to angiotensin-2 by ACE, leading to inflammation, oxidative stress, fibrosis, vasoconstriction and increased vascular permeability contributing to ARDS [71]. In addition, it has been reported that some antihypertensive drugs can affect hemoglobin levels in hypertensive patients with diabetes mellitus, which could be associated with the severity of COVID-19 [70]. In general, comorbidities such as hypertension, diabetes, obesity, and smoking are strongly associated with vascular endothelial damage, dysfunction of the hemostatic system, and chronic inflammation. This can lead to increased cytokines, multiple organ failure (MOF), which can lead to acute respiratory distress syndrome (ARDS) and aggravation of the patient’s condition in case of SARS-CoV-2 infection [71]. According to our estimations, between 8.62 % and 18.47 % of severe cases could have been avoided if the prevalence of hypertension (OR = 2.22; PAF = 14.29 %), coronary heart disease (OR = 2.17; PAF = 8.62 %) and complications such as acute respiratory distress syndrome (ARDS) (OR = 13.09; PAF = 18.47 %) had been reduced. In our meta-analysis, cerebrovascular disease was one of the predominant factors in the severity of COVID-19. Cerebrovascular disease was found to be associated with a 2.76-fold increased risk of severe COVID-19 in patients infected with SARS-CoV-19. However, there was a non-significant trend with cerebrovascular disease in COVID-19 patients in our study. Our results are in agreement with those of Aggarwal and his colleagues [74]. Some studies have shown that comorbidity such as COPD in patients with COVID-19 was one of the potential risk factors associated with the severity of COVID-19 compared with asthmatic/allergic patients [47,52]. In asthmatics, the upper and lower airways are mainly affected, whereas in COPD patients, the altered lesions extend from the small peripheral airways to the alveolar tissues, which can lead to the severe form of coronavirus disease (COVID-19). The lesions caused by COPD are similar to those caused by SARS-CoV-2 and this could increase the risk of severe COVID-19. However, longterm use of inhaled corticosteroids to control asthma may have a beneficial modulatory effect on patients with COVID-19 by reducing epithelial damage and enhancing T-cell immune responses [47,52]. The results of our analysis were in agreement with these related studies, showing that the risk of severe COVID-19 was higher in patients with pre-existing COPD (OR = 1.95; 95 % CI: 1.22–3.09) with a prevalence of 5.35 % in contrast to asthmatic or allergic patients who did not present an increased risk of severe COVID-19 (OR = 1.04; 95 % CI: 0.79–1.36) with a prevalence of 0.27 %. COPD can engender airway obstruction and respiratory complications such as ARDS [69,75,76].

As reported by Fang and his colleagues, among the comorbidities, whether malignancy contributes to the worsening of coronavirus disease remains controversial [51]. Some studies have shown that patients with malignancy did not show an increased risk of developing a severe form of COVID-19 [69,77]. This could be due to the attenuated flow of cytokines caused by their compromised immune systems [69,77]. However, some studies have shown that cancer patients are more likely to develop a severe form of the disease [78]. Our study showed that malignancy was a risk factor for disease worsening (OR = 1.63). Furthermore, we observed a low prevalence of severe COVID-19 in patients with malignancy in our study (PAF = 2.32 %). These results are in agreement with related studies such as Desai and his colleagues and Elgohary and his colleagues [78,79]. However, it is not easy to make strong conclusions for several reasons, such as the small number of cancer patients with a severe form of COVID-19, the lack of information on cancer staging, treatment phase, comorbidities, type of cancer treatment, planned treatment for COVID-19 infection and the ability of the healthcare system to manage these patients [78]. All these factors can influence patient outcomes. Regarding the risk of developing the severe COVID-19 associated with chronic liver disease, we observed an OR of 1.33 with a 95 % CI: 0.82–2.16. Compared with the results of Nagarajan and his colleagues, who found an OR of 2.44 with a 95 % CI: 1.8–3.16 for chronic liver disease, our result suggests a weaker association between chronic liver disease and the risk of developing the severe form of COVID-19 [53]. Furthermore, chronic kidney disease (CKD) (OR = 2.22) and pre-existing acute kidney injury (AKI) (OR = 16.91) were risk factors for disease worsening and hampered recovery in patients with severe COVID-19 compared to patients with nonsevere COVID-19. Previous studies with similar results to ours suggested that it was likely that patients with CKD had a disorder of the immune system, suggesting that CKD and pre-existing AKI complications could be involved in the severe COVID-19 disease [54,69]. Shock (OR = 28.08), AKI (OR = 16.91), ARDS (OR = 13.09), and arrhythmia (OR = 7.47) were principal complications associated with the severity of COVID-19. Al-Mutair and his colleagues reported that excessive lung inflammation, increased pulmonary capillary permeability and alveolar fluid accumulation induced by SARS-CoV-2 were associated with ARDS, for this reason, some ARDS patients required respiratory assistance, particularly diagnosed in patients with severe COVID-19, and this could be due to increased levels of interleukin-6 (IL-6) and tumor necrosis factor alpha (TNF-α) found in these patients [80]. Nevertheless, daily assessment of the Sequential Organ Failure Assessment (SOFA) score and the Multiple Organ Dysfunction (MOD) score associated with septicemia is essential for early diagnosis, treatment and prevention of the consequences of shock and multi-organ dysfunction in patients with severe COVID-19 [80].

Interestingly, the population-attributable risk fractions (PAFs ≥8 %) in this study were statistically significant. Like Li and his colleagues, we found that the highest prevalence of comorbidities was hypertension [69]. However, the prevalence of hypertension in our study (PAF = 14 %) was higher than that reported by Li and his colleagues (PAF = 11.3 %). Among the complications listed in this study, ARDS was the complication contributing to the severity of COVID-19 with a high prevalence in our study (PAF = 18.47 %) compared to the study of Li and his colleagues, in which the complications implicated in the severity of COVID-19 were ARDS (PAF = 10 %) and AKI (PAF = 7.4 %) [69]. Therefore, the risk of severe cases could decrease with the reduction of the prevalence of these factors. In addition, factors such as obesity (PAF= 9.47 %), diabetes (PAF = 9.39 %), and coronary heart disease (PAF=8.62 %) should be controlled. It is important to be cautious about public health guidelines for the management of patients with these comorbidities. However, the other risk factors had PAFs ranging from 0.10 % to 5.78 %, which suggests that these PAFs exerted less effect on the severity of COVID-19 (Table 4).

Our study has several limitations. Eligible studies were limited to articles written in English and French. We were unable to assess the impact of SARS-CoV-2 variants on COVID-19 risk or the effect of vaccination on decreasing severe cases in patients; although all the studies reviewed compared severe and non-severe cases. However, some articles did not specify their definition criteria for severe and non-severe cases, which could bias the results of these studies. Comorbidities and complications can be measured differently from one study to another, which can introduce measurement bias. The quality of the data included in the meta-analysis depends on the quality of the original studies. Unmeasured or unaccounted confounding factors could exist in the included studies, which could influence the results. If some studies have significant methodological flaws, this can affect the validity of the aggregated results.

## 5. Conclusion

In this meta-analysis, the group of patients with severe COVID-19 had a higher prevalence of demographic characteristics, comorbidities, and complications compared to the group of nonsevere COVID-19 patients. However, several risk factors associated with an increased risk of worsening coronavirus disease (COVID-19) have been reported. These included advanced age, sex, obesity, pre-existing smoking, as well as comorbidities such as diabetes, hypertension, malignancy, chronic kidney disease (CKD), chronic obstructive pulmonary disease (COPD), cerebrovascular disease, and coronary artery disease. In addition, complications such as septic shock, acute kidney injury (AKI), acute respiratory distress syndrome (ARDS), and arrhythmia can have lasting consequences on patients’ health, leading to an undesirable disease course. These outcomes are crucial in guiding timely treatment decisions and assessing disease prognosis.

## Support information

S1 PRISMA 2020 Checklist.



## Figures and Tables

**Table 1:** Clinical and demographic characteristics of the patients with severe and non-severe COVID-19.

Reference	Publication year	Country	Samples (Male/Female)	Severe	Non-severe	Overall age (Median age/Mean age, range/SD)	Risk factor outcomes
Al-Numair et al., 2022 [14]	2022	Saudi Arabia	598 (352/246)	300	298	57 (46–65)	➀, ➁, ➃, ➆, ➈, ⑫, ⑭, ⑮
Almarashda et al., 2022 [15]	2022	United Arab Emirates	585 (386/199)	154	431	49 (39–59)	➀, ➁, ➂, ➃, ➅, ➆, ➈, ⑪, ⑮
Cai et al., 2020 [16]	2020	China	298 (145/153)	58	240	47,5 (33–61)	➀, ➁, ➃, ➆, ⑫
Chen et al., 2023 [17]	2023	China (Taiwan)	127 (70/57)	39	88	52,3 (SD = 14.1)	➀, ➁, ➃, ➆, ⑫
El Aidaoui et al., 2020 [18]	2020	Morocco	134 (73/61)	45	89	53 (36–64)	➀, ➁, ➂, ➃, ➄, ➅, ➆, ➉, ⑪, ⑫, ⑬, ⑭, ⑯
Feng et al., 2020 [19]	2020	China	476 (271/205)	124	352	53 (40–64)	➀, ➁, ➂, ➃, ➄, ➅, ➆, ➇, ➈, ⑪
Hermosa et al., 2023 [20]	2023	Spain	2217 (1210/1007)	872	1145	62.2 (SD = 12.8)	➀, ➁, ➂, ➃, ➄, ➅, ➆, ➈, ⑫, ⑯
Hong et al., 2020 [21]	2020	Korea	98 (38/60)	13	85	55.4 (SD = 17.1)	➀, ➁, ➃, ➄, ➅, ➆, ➇, ➈, ➉, ⑫, ⑬, ⑭
Ishii et al., 2020 [22]	2020	Japan	345 (198/147)	112	233	54 (32–68)	➀, ➁, ➃, ➄, ➅, ➆, ➈, ⑪, ⑫
Kabuto et al., 2023 [23]	2023	Italy	192 (138/54)	44	148	68.5 (65.2–71.8)	➀, ➁, ➂, ➃, ➆, ➈, ⑭, ⑮
Kantri et al., 2021 [24]	2021	Morocco	138 (73/61)	45	89	53 (36–64)	➀, ➁, ➂, ➃, ➄, ➅, ➆, ➉, ⑪, ⑫, ⑭, ⑯
Lenoir et al., 2023 [25]	2023	Swiss	584 (332/252)	228	356	58 (SD = 14.1)	➀, ➁, ➂, ➃, ➄, ➅, ➆, ➈, ⑪, ⑭, ⑯
Namayandeh et al., 2023 [26]	2023	Iran	19,576 (10,291/9285)	2559	17,017	56.2 (SD = 20.71)	➀, ➁, ➂, ➃, ➄, ➅, ➆, ➈, ⑪, ⑫, ⑯
Ozel et al., 2022 [27]	2022	Turkey	728 (392/336)	37	691	54 (44–65)	➀, ➁, ➃, ➄, ➅, ➆, ➈, ⑪, ⑫, ⑭, ⑯
Pecorelli et al., 2023 [28]	2023	Switzerland	168 (125/43)	91	77	63 (SD = 12)	➀, ➁, ➃, ➄, ➅, ➆, ➇, ➈, ⑪, ⑯
Ren et al., 2020 [29]	2020	China	129 (62/67)	40	89	50 (34.5–61)	➀, ➁, ➃, ➄, ➅, ➆, ⑪
Reyes et al., 2022a [30]	2022	Colombia	40,440 (25,501/14,939)	20,044	20,396	67 (55–78)	➀, ➁, ➃, ➄, ➅, ➆, ➈, ⑪, ⑫, ⑭, ⑮, ⑯
Reyes et al., 2022b [31]	2022	Colombia	3008 (1817/1191)	1934	1074	56 (43–67)	➀, ➁, ➂, ➃, ➄, ➅, ➆, ➇, ➈, ⑪, ⑭, ⑯
Ryan et al., 2021 [32]	2021	USA	556 (296/260)	164	392	57 (SD = 17)	➀, ➁, ➂, ➃, ➄, ➅, ➆, ➇, ➈, ⑪, ⑫, ⑮, ⑯
Shu et al., 2020 [33]	2020	China	293 (135/158)	86	207	57.1 (SD = 16.3)	➀, ➁, ➃, ➅, ➆, ➇, ⑪
Su et al., 2023 [34]	2023	Canada	417 (217/200)	175	242	65.3 (18.4)	➀, ➁, ➂, ➃, ➅, ➆, ➈, ⑪, ⑫
Suleyman et al., 2020 [35]	2020	USA	463 (165/298)	141	214	57.5 (SD = 16.8)	➀, ➁, ➂, ➃, ➅, ➆, ➈, ⑪, ⑬, ⑮, ⑯
Takács et al., 2023 [36]	2023	Hungary	83 (53/30)	32	51	70.1 (SD = 12.8)	➀, ➁, ➃, ➅, ➆, ➈
Uzum et al., 2023 [37]	2023	Turkey	272 (144/128)	114	158	65 (SD = 14)	➀, ➁, ➃, ➃, ➅, ➆, ⑪
Wang et al., 2020 [38]	2020	China	138 (75/102)	36	102	56 (42–68)	➀, ➁, ➃, ➅, ➆, ➇, ➈, ➉, ⑪, ⑫, ⑬, ⑭
Wei et al., 2020 [39]	2020	China	276 (155/121)	14	262	51 (41–58)	➀, ➁, ➂, ➃, ➅, ➆, ➇, ➈
Xiong et al., 2020 [40]	2020	China	116 (80/36)	55	61	58.5 (47–69)	➀, ➁, ➃, ➅, ➆, ➇, ➈, ➉, ⑫, ⑬, ⑭
Xu et al., 2023 [41]	2023	China	11,566 (6959/4607)	2422	9144	65.45 (SD = 15.61)	➀, ➁, ➄, ➅, ➆, ➈, ➉, ⑫, ⑮
Yahyaoui et al., 2023 [42]	2023	Morocco	521 (272/249)	185	336	64 (SD = 16.3)	➀, ➁, ➃, ➅, ➆, ➈, ⑪, ⑮
Yang et al., 2021 [43]	2021	China	78 (37/41)	17	61	45.5 (34–55.8)	➀, ➁, ➂, ➃, ➅, ➆, ⑭
Yousif et al., 2022 [44]	2022	Sudan	418 (279/139)	279	139	66,3 (SD = 13)	➀, ➁, ➂, ➃, ➅, ➆, ➇, ➈, ⑪
Zhichao Feng et al., 2020 [45]	2020	China	564 (284/280)	69	495	47 (36–58)	➀, ➁, ➂, ➃, ➅, ➆, ➇, ➈, ⑪, ⑫, ⑮
Zhou et al., 2021 [46]	2021	China	120 (49/71)	16	104	51.6 (SD = 10.8)	➀, ➁, ➂, ➃, ➅, ➆

➀: sex; ➁: age; ➂: smoking history; ➃: hypertension; ➄: malignancy; ➅: coronary heart disease; ➆: diabetes; ➇: cerebrovascular disease; ➈: chronic obstructive pulmonary disease (COPD); ➉: shock; ⑪: chronic kidney disease (CKD); ⑫: chronic liver disease; ⑬: acute kidney injury (AKI); ⑭: acute respiratory distress syndrome (ARDS); ⑮: body mass index (BMI); ⑯: asthma/Allergy.

**Table 2:** Evaluation of the methodological quality of studies according to the NOS score.

Study	Selection				Comparability	Outcome			Total score
		
	Representativeness of the exposed cohort	Selection of the nonexposed cohort	Ascertainment of exposure	Demonstration that outcome of interest was not present at start of study	Comparability of cohorts on the basis of the design or analysis	Assessment of outcome	Followup time	Adequacy of follow-up of cohorts	
Al-Numair et al., 2022 [14]	*	*	*	*	**	*	*	*	9
Almarashda et al., 2022 [15]	*	*	*	*	**	*		*	8
Cai et al., 2020 [16]	*	*	*	*	**	*	*	*	9
Chen et al., 2023 [17]	*	*	*	*	*			*	6
El Aidaoui et al., 2020 [18]	*	*	*	*	*	*	*	*	8
Feng et al., 2020 [19]	*	*	*	*	**	*	*	*	9
Hermosa et al., 2023 [20]	*	*	*	*	**	*	*		7
Hong et al., 2020 [21]	*	*	*	*	*	*	*		7
Ishii et al., 2020 [22]	*	*	*	*	**	*	*	*	9
Kabuto et al., 2023 [23]	*	*	*	*	**	*	*	*	9
Kantri et al., 2021 [24]	*	*	*	*	**	*	*	*	9
Lenoir et al., 2023 [25]	*	*	*	*	**	*	*	*	9
Namayandeh et al., 2023 [26]	*	*	*	*	*			*	6
Ozel et al., 2022 [27]	*	*	*	*	**	*	*	*	9
Pecorelli et al., 2023 [28]	*	*	*	*	*	*		*	7
Ren et al., 2020 [29]	*	*	*	*	**	*	*	*	9
Reyes et al., 2022a [30]	*	*	*	*	**	*		*	8
Reyes et al., 2022b [31]	*	*	*	*	*	*		*	7
Ryan et al., 2021 [32]	*	*	*	*	**	*	*	*	9
Shu et al., 2020 [33]	*	*	*	*	**	*	*	*	9
Su et al., 2023 [34]	*	*	*	*	*	*		*	7
Suleyman et al., 2020 [35]	*	*	*	*	**	*	*	*	9
Takács et al., 2023 [36]	*	*	*	*	*	*		*	7
Uzum et al., 2023 [37]	*	*	*	*	*		*	*	7
Wang et al., 2020 [38]	*	*	*	*	*	*	*	*	8
Wei et al., 2020 [39]	*	*	*	*	**	*	*	*	9
Xiong et al., 2020 [40]	*	*	*	*	*	*	*	*	8
Xu et al., 2023 [41]	*	*	*	*	*	*	*	*	8
Yahyaoui et al., 2023 [42]	*	*	*	*	**	*		*	8
Yang et al., 2021 [43]	*	*	*	*	*	*		*	7
Yousif et al., 2022 [44]	*	*	*	*	**	*		*	8
Zhichao Feng et al., 2020 [45]	*	*	*	*	**	*		*	8
Zhou et al., 2021 [46]	*	*	*	*	*		*	*	7

**Table 3:** Results of meta-analysis for primary outcomes.

Outcome or Subgroup	Studies	Participants	Statistical Method	Effect Estimate	I^2^
(a) Sex	33	85,812	Odds Ratio (M–H, Random, 95 % CI)	1.52 [1.34, 1.73]	85 %
(b) Age	28	24,168	Std. Mean Difference (IV, Random, 95 % CI)	0.62 [0.43, 0.82]	99 %
(c) Asthma/allergy	13	68,323	Odds Ratio (M–H, Random, 95 % CI)	1.04 [0.79, 1.36]	71 %
(d) Body mass index (BMI)	10	58,388	Odds Ratio (M–H, Random, 95 % CI)	2.11 [1.47, 3.04]	96 %
(e) Smoking history	17	29,870	Odds Ratio (M–H, Random, 95 % CI)	1.33 [1.01, 1.75]	67 %
(f) Diabetes	33	85,812	Odds Ratio (M–H, Random, 95 % CI)	1.81 [1.35, 2.43]	97 %
(g) Hypertension	32	74,248	Odds Ratio (M–H, Random, 95 % CI)	2.22 [1.72, 2.87]	96 %
(h) Malignancy	22	81,894	Odds Ratio (M–H, Random, 95 % CI)	1.63 [1.07, 2.49]	94 %
(i) Chronic kidney disease (CKD)	21	70,243	Odds Ratio (M–H, Random, 95 % CI)	2.27 [1.26, 4.06]	96 %
(j) Chronic obstructive pulmonary disease (COPD)	24	84,227	Odds Ratio (M–H, Random, 95 % CI)	1.95 [1.22, 3.09]	98 %
(k) Cerebrovascular disease	11	6111	Odds Ratio (M–H, Random, 95 % CI)	2.76 [1.65, 4.62]	44 %
(l) Coronary heart disease	29	84,597	Odds Ratio (M–H, Random, 95 % CI)	2.17 [1.42, 3.31]	98 %
(m) Chronic liver disease	17	78,254	Odds Ratio (M–H, Random, 95 % CI)	1.33 [0.82, 2.16]	91 %
(n) Shock	7	12,541	Odds Ratio (M–H, Random, 95 % CI)	28.08 [3.49, 226.03]	86 %
(o) Acute kidney injury (AKI)	4	397	Odds Ratio (M–H, Random, 95 % CI)	16.91 [1.87, 152.45]	63 %
(p) Acute respiratory distress syndrome (ARDS)	12	46,248	Odds Ratio (M–H, Random, 95 % CI)	13.09 [5.87, 29.18]	94 %
(q) Neurological complications	6	64,504	Odds Ratio (M–H, Random, 95 % CI)	0.95 [0.35, 2.64]	97 %
(r) Arrhythmia	5	41,444	Odds Ratio (M–H, Random, 95 % CI)	7.47 [2.96, 18.83]	34 %

**Table 4:** Proportion of risk attributable to different risk factors for severe forms of the disease.

Outcome or Subgroup	Prevalence	PAF	OR (95 % CI)
(c) Asthma/allergy	0.07	0.27 %	1.04 [0.79, 1.36]
(d) Body mass index (BMI)	0.18	9.47 %	2.11 [1.47, 3.04]
(e) Smoking history	0.05	1.24 %	1.33 [1.01, 1.75]
(f) Diabetes	0.21	9.39 %	1.81 [1.35, 2.43]
(g) Hypertension	0.26	14.29 %	2.22 [1.72, 2.87]
(h) Malignancy	0.06	2.32 %	1.63 [1.07, 2.49]
(i) Chronic kidney disease (CKD)	0.07	4 %	2.27 [1.26, 4.06]
(j) Chronic obstructive pulmonary disease (COPD)	0.11	5.35 %	1.95 [1.22, 3.09]
(k) Cerebrovascular disease	0.06	3.82 %	2.76 [1.65, 4.62]
(l) Coronary heart disease	0.16	8.62 %	2.17 [1.42, 3.31]
(m) Chronic liver disease	0.04	0.10 %	1.33 [0.82, 2.16]
(n) Shock	0.06	5.78 %	28.08 [3.49, 226.03]
(o) Acute kidney injury (AKI)	0.06	5.64 %	16.91 [1.87, 152.45]
(p) Acute respiratory distress syndrome (ARDS)	0.20	18.47 %	13.09 [5.87, 29.18]
(r) Arrhythmia	0.002	0.17 %	7.47 [2.96, 18.83]

## References

[1] Bean J Kuri-Cervantes L Pennella M Betts MR Meyer NJ Hassan WM Multivariate indicators of disease severity in COVID-19 Sci Rep 2023 13 5145 10.1038/s41598-023-31683-9 36991002 PMC10054197

[2] Zhang J Dong X Liu G Gao Y Risk and Protective factors for COVID-19 morbidity, severity, and mortality Clin Rev Allergy Immunol 2023 64 90 107 10.1007/s12016-022-08921-5 35044620 PMC8767775

[3] de Bruin S Bos LD van Roon MA Tuip-de Boer AM Schuurman AR Koel-Simmelinck MJA Clinical features and prognostic factors in Covid-19: a prospective cohort study EBioMedicine 2021 67 103378 10.1016/j.ebiom.2021.103378 34000622 PMC8118723

[4] Arena R Pronk NP Laddu D Whitsel LP Sallis JF Lavie CJ Mapping one million COVID-19 deaths and unhealthy lifestyle behaviors in the United States: recognizing the syndemic pattern and taking action Am J Med 2022 135 1288 95 10.1016/j.amjmed.2022.06.006 35820461 PMC9270235

[5] Arena R Lavie CJ The global path forward – healthy living for pandemic event protection (HL – PIVOT) Prog Cardiovasc Dis 2021 64 96 101 10.1016/j.pcad.2020.05.008 32485186 PMC7834504

[6] Arena R Use of the healthy living medicine platform to minimize COVID-19 vaccination: dispelling this myth before it takes hold Am J Med 2023 136 403 4 10.1016/j.amjmed.2022.12.035 36649840 PMC9840224

[7] Gao Y Ding M Dong X Zhang J Kursat Azkur A Azkur D Risk factors for severe and critically ill COVID-19 patients: a review Allergy 2021 76 428 55 10.1111/all.14657 33185910

[8] Lee SW Koo MJ PRISMA 2020 statement and guidelines for systematic review and meta-analysis articles, and their underlying mathematics: life Cycle Committee Recommendations Life Cycle 2022 2 10.54724/lc.2022.e9

[9] Stroup DF Berlin JA Morton SC Olkin I Williamson GD Rennie D Meta-analysis of observational studies in epidemiology: a proposal for reporting. Meta-analysis of Observational Studies in Epidemiology (MOOSE) group JAMA 2000 283 2008 12 10.1001/jama.283.15.2008 10789670

[10] Wells GA Shea B Ottawa hospital research institute https://www.ohri.ca/programs/clinical_epidemiology/oxford.asp accessed June 8, 2023

[11] Collaboration C Review manager (RevMan)[program] Cph Nord Cochrane Cent Cochrane Collab 2014 https://training.cochrane.org/online-learning/core-software/revman accessed 8 April 2023

[12] Miettinen OS Proportion of disease caused or prevented by a given exposure, trait or intervention Am J Epidemiol 1974 99 325 32 10.1093/oxfordjournals.aje.a121617 4825599

[13] Murad MH Wang Z Chu H Lin L When continuous outcomes are measured using different scales: guide for metaanalysis and interpretation BMJ 2019 364 k4817 10.1136/bmj.k4817 30670455 PMC6890471

[14] Al-Numair NS Alyounes B Al-Saud H Halwani R Al-Muhsen S Clinical characteristics, risk factors, and rate of severity of a nationwide COVID-19 Saudi cohort Saudi J Biol Sci 2022 29 103315 10.1016/j.sjbs.2022.103315 35645590 PMC9124585

[15] Almarashda AMJ Rabbani SA Kurian MT Cherian A Clinical characteristics, risk factors for severity and pharmacotherapy in hospitalized COVID-19 patients in the United Arab Emirates J Clin Med 2022 11 2439 10.3390/jcm11092439 35566563 PMC9100822

[16] Cai Q Huang D Ou P Yu H Zhu Z Xia Z COVID-19 in a designated infectious diseases hospital outside Hubei Province, China Allergy 2020 75 1742 52 10.1111/all.14309 32239761

[17] Chen PK Yeo KJ Chang SH Liao TL Chou CH Lan JL The detectable antiinterferon-γ autoantibodies in COVID-19 patients may be associated with disease severity Virol J 2023 20 33 10.1186/s12985-023-01989-1 36810114 PMC9942050

[18] El Aidaoui KE Haoudar A Khalis M Kantri A Ziati J Ghanmi AE Predictors of severity in Covid-19 patients in casablanca Morocco Cureus 2020 12 10.7759/cureus.10716 PMC7532862 33033687

[19] Feng Y Ling Y Bai T Xie Y Huang J Li J COVID-19 with different severities: a multicenter study of clinical features Am J Respir Crit Care Med 2020 201 1380 8 10.1164/rccm.202002-0445OC 32275452 PMC7258639

[20] Hermosa JLR Vargas Centanaro G González Castro ME Miravitlles M LázaroAsegurado L Jiménez-Rodríguez BM Severe COVID-19 illness and α1-antitrypsin deficiency: COVID-AATD study Biomedicines 2023 11 516 10.3390/biomedicines11020516 36831051 PMC9953718

[21] Hong KS Lee KH Chung JH Shin KC Choi EY Jin HJ Clinical features and outcomes of 98 patients hospitalized with SARS-CoV-2 infection in Daegu, South Korea: a brief descriptive study Yonsei Med J 2020 61 431 7 10.3349/ymj.2020.61.5.431 32390367 PMC7214108

[22] Ishii M Terai H Kabata H Masaki K Chubachi S Tateno H Clinical characteristics of 345 patients with coronavirus disease 2019 in Japan: a multicenter retrospective study J Infect 2020 81 e3 5 10.1016/j.jinf.2020.08.052 32920063 PMC7482596

[23] Kabuto T Seo R Miyakoshi C Shimizu Y Shima Y Yamashita D Time dependency and unique etiology of barotrauma in COVID-19: a retrospective cohort study with landmark analysis and pathological approach PLoS One 2023 18 e0282868 10.1371/journal.pone.0282868 36921007 PMC10016681

[24] Kantri A Ziati J Khalis M Haoudar A Aidaoui KE Daoudi Y Hematological and biochemical abnormalities associated with severe forms of COVID-19: a retrospective single-center study from Morocco PLoS One 2021 16 e0246295 10.1371/journal.pone.0246295 33539383 PMC7861397

[25] Lenoir A Christe A Ebner L Beigelman-Aubry C Bridevaux P-O Brutsche M Pulmonary recovery 12 Months after non-severe and severe COVID-19: the prospective Swiss COVID-19 lung study Respiration 2023 102 120 33 10.1159/000528611 36566741 PMC9932828

[26] Namayandeh SM Dehghan H Lotfi MH Khajehaminian MR Hosseini S Bahrevar V Clinical courses of 24,563 hospitalized COVID-19 patients during the first 12 months of the pandemic in the Central City of Iran Sci Rep 2023 13 6521 10.1038/s41598-023-32292-2 37085530 PMC10119518

[27] Ozel AS Altunal LN Aydin M Unal B Cam G Ozer MC Clinical characteristics and risk factors associated with severe disease and outcome of patients with COVID-19 J Infect Dev Ctries 2022 16 435 44 10.3855/jidc.15411 35404848

[28] Pecorelli N Eggmann S Jeitziner MM Que YA Messmer AS Early rehabilitation interventions and physical therapy in adults who were critically ill with COVID-19 pneumonia: a retrospective observational study Phys Ther 2023 103 pzac157 10.1093/ptj/pzac157 37104624

[29] Ren L Yao D Cui Z Chen S Yan H Corona Virus Disease 2019 patients with different disease severity or age range Medicine (Baltim) 2020 99 e22899 10.1097/MD.0000000000022899 PMC7717834 33285678

[30] Reyes LF Murthy S Garcia-Gallo E Irvine M Merson L Martin-Loeches I Clinical characteristics, risk factors and outcomes in patients with severe COVID-19 registered in the International Severe Acute Respiratory and Emerging Infection Consortium WHO clinical characterisation protocol: a prospective, multinational, multicentre, observational study ERJ Open Res 2022 8 552 2021 10.1183/23120541.005522021 PMC8669808 35169585

[31] Reyes LF Bastidas A Narváez PO Parra-Tanoux D Fuentes YV Serrano-Mayorga CC Clinical characteristics, systemic complications, and in-hospital outcomes for patients with COVID-19 in Latin America. LIVEN-Covid-19 study: a prospective, multicenter, multinational, cohort study PLoS One 2022 17 e0265529 10.1371/journal.pone.0265529 35358238 PMC8970353

[32] Ryan C Minc A Caceres J Balsalobre A Dixit A Ng BK Predicting severe outcomes in Covid-19 related illness using only patient demographics, comorbidities and symptoms Am J Emerg Med 2021 45 378 84 10.1016/j.ajem.2020.09.017 33046294 PMC7480533

[33] Shu Z Zhou Y Chang K Liu J Min X Zhang Q Clinical features and the traditional Chinese medicine therapeutic characteristics of 293 COVID-19 inpatient cases Front Med 2020 14 760 75 10.1007/s11684-020-0803-8 32926319 PMC7488634

[34] Su C-Y Zhou S Gonzalez-Kozlova E Butler-Laporte G Brunet-Ratnasingham E Nakanishi T Circulating proteins to predict COVID-19 severity Sci Rep 2023 13 6236 10.1038/s41598-023-31850-y 37069249 PMC10107586

[35] Suleyman G Fadel RA Malette KM Hammond C Abdulla H Entz A Clinical characteristics and morbidity associated with coronavirus disease 2019 in a series of patients in metropolitan detroit JAMA Netw Open 2020 3 e2012270 10.1001/jamanetworkopen.2020.12270 32543702 PMC7298606

[36] Takács TT Berki Áj Böjti PP Stang R Fritz-Reunes PA Schnekenberg L The impact of SARS-CoV-2 infection on the outcome of acute ischemic stroke—a retrospective cohort study PLoS One 2023 18 e0282045 10.1371/journal.pone.0282045 36862706 PMC9980769

[37] Uzum Y Turkkan E Uzum Y Turkkan E Predictivity of CRP, albumin, and CRP to albumin ratio on the development of intensive care requirement, mortality, and disease severity in COVID-19 Cureus 2023 15 10.7759/cureus.33600 PMC9910810 36788868

[38] Wang D Hu B Hu C Zhu F Liu X Zhang J Clinical characteristics of 138 hospitalized patients with 2019 novel coronavirus–infected pneumonia in wuhan, China JAMA 2020 323 1061 9 10.1001/jama.2020.1585 32031570 PMC7042881

[39] Wei Y Zeng W Huang X Li J Qiu X Li H Clinical characteristics of 276 hospitalized patients with coronavirus disease 2019 in Zengdu District, Hubei Province: a single-center descriptive study BMC Infect Dis 2020 20 549 10.1186/s12879-020-05252-8 32727456 PMC7388483

[40] Xiong S Liu L Lin F Shi J Han L Liu H Clinical characteristics of 116 hospitalized patients with COVID-19 in Wuhan, China: a single-centered, retrospective, observational study BMC Infect Dis 2020 20 787 10.1186/s12879-02005452-2 33092539 PMC7578439

[41] Xu C Zheng L Jiang Y Jin L A prediction model for predicting the risk of acute respiratory distress syndrome in sepsis patients: a retrospective cohort study BMC Pulm Med 2023 23 78 10.1186/s12890-023-02365-z 36890503 PMC9994387

[42] Yahyaoui A Amrani A Idrissi A Belmahi S Nassiri O Mouhoub B Comparative analysis of clinical and biological characteristics of COVID-19 patients: a retrospective cohort study Clin Epidemiol Glob Health 2023 19 101184 10.1016/j.cegh.2022.101184 36447933 PMC9691507

[43] Yang H Xu Y Li Z Yan L Wang J Liao P The clinical implication of dynamic hematological parameters in COVID-19: a retrospective study in chongqing, China Int J Gen Med 2021 14 4073 80 10.2147/IJGM.S321292 34354369 PMC8331199

[44] Yousif MYE Eljack MMFA Haroun MS Abbasher Hussien Mohamed Ahmed K Amir O Alfatih M Clinical characteristics and risk factors associated with severe disease progression among COVID-19 patients in wad medani isolation centers: a multicenter retrospective cross-sectional study Health Sci Rep 2022 5 e523 10.1002/hsr2.523 35284652 PMC8900979

[45] Feng Z Li J Yao S Yu Q Zhou W Mao X Clinical factors associated with progression and prolonged viral shedding in COVID-19 patients: a multicenter study Aging Dis 2020 11 1069 81 10.14336/AD.2020.0630 33014523 PMC7505267

[46] Zhou F Tao M Shang L Liu Y Pan G Jin Y Assessment of sequelae of COVID19 nearly 1 Year after diagnosis Front Med 2021 8 10.3389/fmed.2021.717194 PMC8649686 34888318

[47] Chenchula S Vidyasagar K Pathan S Sharma S Chavan MR Bhagavathula AS Global prevalence and effect of comorbidities and smoking status on severity and mortality of COVID-19 in association with age and gender: a systematic review, metaanalysis and meta-regression Sci Rep 2023 13 6415 10.1038/s41598-02333314-9 37076543 PMC10115382

[48] Liu H Chen S Liu M Nie H Lu H Comorbid chronic diseases are strongly correlated with disease severity among COVID-19 patients: a systematic review and MetaAnalysis Aging Dis 2020 11 668 78 10.14336/AD.2020.0502 32489711 PMC7220287

[49] Zhang J Dong X Liu G Gao Y Risk and protective factors for COVID-19 morbidity, severity, and mortality Clin Rev Allergy Immunol 2023 64 90 107 10.1007/s12016-022-08921-5 35044620 PMC8767775

[50] Djorwé S Bousfiha A Nzoyikorera N Nkurunziza V Ait Mouss K Kawthar B Epidemiology, clinical characteristics and risk factors of coronavirus disease 2019 (COVID-19) in Casablanca Access Microbiol 2023 5 000400 10.1099/acmi.0.000400 PMC10202397 37223059

[51] Fang X Li S Yu H Wang P Zhang Y Chen Z Epidemiological, comorbidity factors with severity and prognosis of COVID-19: a systematic review and metaanalysis Aging 2020 12 12493 503 10.18632/aging.103579 32658868 PMC7377860

[52] Uruma Y Manabe T Fujikura Y Iikura M Hojo M Kudo K Effect of asthma, COPD, and ACO on COVID-19: a systematic review and meta-analysis PLoS One 2022 17 e0276774 10.1371/journal.pone.0276774 36318528 PMC9624422

[53] Nagarajan R Krishnamoorthy Y Rajaa S Hariharan VS COVID-19 severity and mortality among chronic liver disease patients: a systematic review and MetaAnalysis Prev Chronic Dis 2022 19 E53 10.5888/pcd19.210228 36007255 PMC9480842

[54] Liu YF Zhang Z Pan XL Xing GL Zhang Y Liu ZS The chronic kidney disease and acute kidney injury involvement in COVID-19 pandemic: a systematic review and meta-analysis PLoS One 2021 16 e0244779 10.1371/journal.pone.0244779 33400721 PMC7785235

[55] Aleksanyan Y Weinman JP Women, men and COVID-19 Soc Sci Med 1982 2022 294 114698 10.1016/j.socscimed.2022.114698 PMC8732878 34999529

[56] Gallus S Scala M Possenti I Jarach CM Clancy L Fernandez E The role of smoking in COVID-19 progression: a comprehensive meta-analysis Eur Respir Rev 2023 32 10.1183/16000617.0191-2022 PMC10032583 36889786

[57] Ghosh S Klein RS Sex drives dimorphic immune responses to viral infections J Immunol 2017 198 1782 90 10.4049/jimmunol.1601166 28223406 PMC5325721

[58] Pirhadi R Sinai Talaulikar V Onwude J Manyonda I Could estrogen protect women from COVID-19? J Clin Med Res 2020 12 634 9 10.14740/jocmr4303 33029269 PMC7524561

[59] Goren A McCoy J Wambier CG Vano-Galvan S Shapiro J Dhurat R What does androgenetic alopecia have to do with COVID-19? An insight into a potential new therapy Dermatol Ther 2020 33 e13365 10.1111/dth.13365 32237190 PMC7228378

[60] Shieh WJ Hsiao CH Paddock CD Guarner J Goldsmith CS Tatti K Immunohistochemical, in situ hybridization, and ultrastructural localization of SARSassociated coronavirus in lung of a fatal case of severe acute respiratory syndrome in Taiwan Hum Pathol 2005 36 303 9 10.1016/j.humpath.2004.11.006 15791576 PMC7112064

[61] Dalpiaz PLM Lamas AZ Caliman IF Ribeiro RF Abreu GR Moyses MR Sex hormones promote opposite effects on ACE and ACE2 activity, hypertrophy and cardiac contractility in spontaneously hypertensive rats PLoS One 2015 10 e0127515 10.1371/journal.pone.0127515 26010093 PMC4444272

[62] Lin B Ferguson C White JT Wang S Vessella R True LD Prostate-localized and androgen-regulated expression of the membrane-bound serine protease TMPRSS2 Cancer Res 1999 59 4180 4 10485450

[63] Mikkonen L Pihlajamaa P Sahu B Zhang FP Jänne OA Androgen receptor and androgen-dependent gene expression in lung Mol Cell Endocrinol 2010 317 14 24 10.1016/j.mce.2009.12.022 20035825

[64] Kastora S Patel M Carter B Delibegovic M Myint PK Impact of diabetes on COVID19 mortality and hospital outcomes from a global perspective: an umbrella systematic review and meta-analysis Endocrinol Diabetes Metab 2022 5 e00338 10.1002/edm2.338 35441801 PMC9094465

[65] Zhang T Mei Q Zhang Z Walline JH Liu Y Zhu H Risk for newly diagnosed diabetes after COVID-19: a systematic review and meta-analysis BMC Med 2022 20 444 10.1186/s12916-022-02656-y 36380329 PMC9666960

[66] Yang J Zheng Y Gou X Pu K Chen Z Guo Q Prevalence of comorbidities and its effects in patients infected with SARS-CoV-2: a systematic review and meta-analysis Int J Infect Dis 2020 94 91 5 10.1016/j.ijid.2020.03.017 32173574 PMC7194638

[67] Schlesinger S Lang A Christodoulou N Linnerz P Pafili K Kuss O Risk phenotypes of diabetes and association with COVID-19 severity and death: an update of a living systematic review and meta-analysis Diabetologia 2023 66 1395 412 10.1007/s00125-023-05928-1 37204441 PMC10198038

[68] Badawi A Ryoo SG Prevalence of comorbidities in the Middle East respiratory syndrome coronavirus (MERS-CoV): a systematic review and meta-analysis Int J Infect Dis 2016 49 129 33 10.1016/j.ijid.2016.06.015 27352628 PMC7110556

[69] Li X Zhong X Wang Y Zeng X Luo T Liu Q Clinical determinants of the severity of COVID-19: a systematic review and meta-analysis PLoS One 2021 16 e0250602 10.1371/journal.pone.0250602 33939733 PMC8092779

[70] Nakhaie S Yazdani R Shakibi M Torabian S Pezeshki S Bazrafshani MS The effects of antihypertensive medications on severity and outcomes of hypertensive patients with COVID-19 J Hum Hypertens 2022 1 8 10.1038/s41371-022-00716-7 PMC9255835 35790875

[71] Cinaud A Sorbets E Blachier V Vallee A Kretz S Lelong H Hypertension artérielle et COVID-19 Presse Médicale Form 2021 2 25 32 10.1016/j.lpmfor.2020.08.006

[72] Forouzanfar MH Liu P Roth GA Ng M Biryukov S Marczak L Global burden of hypertension and systolic blood pressure of at least 110 to 115 mm Hg, 1990-2015 JAMA 2017 317 165 82 10.1001/jama.2016.19043 28097354

[73] Madjid M Safavi-Naeini P Solomon SD Vardeny O Potential effects of coronaviruses on the cardiovascular system: a review JAMA Cardiol 2020 5 831 40 10.1001/jamacardio.2020.1286 32219363

[74] Aggarwal G Lippi G Michael Henry B Cerebrovascular disease is associated with an increased disease severity in patients with Coronavirus Disease 2019 (COVID-19): a pooled analysis of published literature Int J Stroke 2020 15 385 9 10.1177/1747493020921664 32310015

[75] Russell R Covid-19 and COPD: a personal reflection Int J Chronic Obstr Pulm Dis 2020 15 883 4 10.2147/COPD.S255101 PMC7188202 32425514

[76] Halpin DMG Rabe AP Loke WJ Grieve S Daniele P Hwang S Epidemiology, healthcare resource utilization, and mortality of asthma and COPD in COVID-19: a systematic literature review and meta-analyses J Asthma Allergy 2022 15 811 25 10.2147/JAA.S360985 35747745 PMC9211747

[77] Miyashita H Mikami T Chopra N Yamada T Chernyavsky S Rizk D Do patients with cancer have a poorer prognosis of COVID-19? An experience in New York City Ann Oncol 2020 31 1088 9 10.1016/j.annonc.2020.04.006 32330541 PMC7172785

[78] ElGohary GM Hashmi S Styczynski J Kharfan-Dabaja MA Alblooshi RM de la Cámara R The risk and prognosis of COVID-19 infection in cancer patients: a systematic review and meta-analysis Hematol Oncol Stem Cell Ther 2020 10.1016/j.hemonc.2020.07.005 PMC7390725 32745466

[79] Desai A Sachdeva S Parekh T Desai R COVID-19 and cancer: lessons from a pooled meta-analysis JCO Glob Oncol 2020 557 9 10.1200/GO.20.00097 32250659 PMC7193801

[80] Al Mutair A Al Mutairi A Zaidi ARZ Salih S Alhumaid S Rabaan AA Clinical predictors of COVID-19 mortality among patients in intensive care units: a retrospective study Int J Gen Med 2021 14 3719 28 10.2147/IJGM.S313757 34321917 PMC8313378

