# Supplementary figures and images for: Impact and prevalence of comorbidities and complications on the severity of COVID-19 in association with age, gender, obesity, and pre-existing smoking: A meta-analysis
Source: Biomedicine (Taipei). 2024 Mar 1;14(1):20–38. doi: 10.37796/2211-8039.1429 (PMC10962561; doi:10.37796/2211-8039.1429)

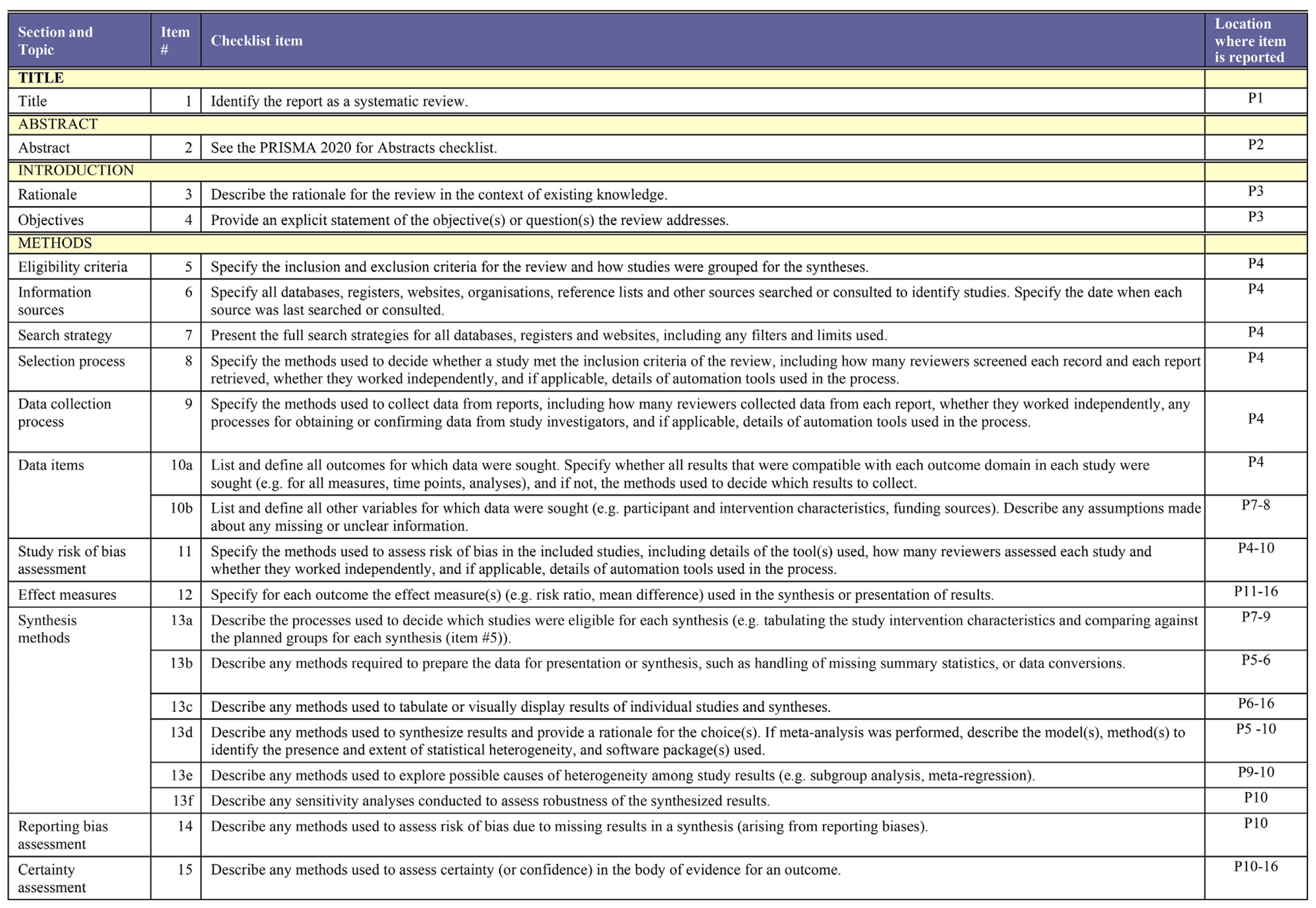

Supplement: Supplementary file 1 [file bmed-14-01-020s1a.tif]

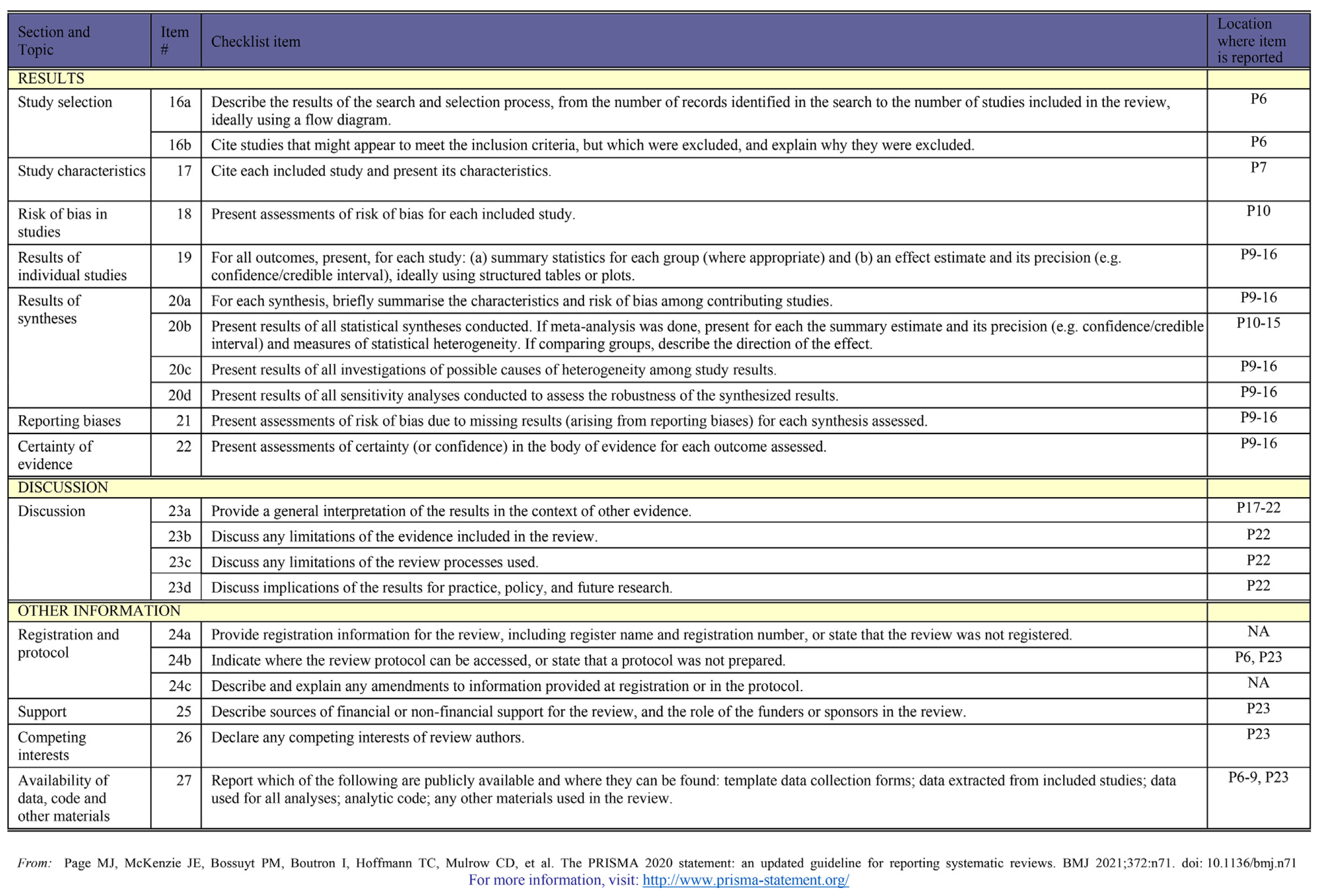

Supplement: Supplementary file 2 [file bmed-14-01-020s1b.tif]
